# Successful Treatment of Critically Severe Empyema With Bronchopleural Fistula via ECMO: A Case Report

**DOI:** 10.1002/rcr2.70162

**Published:** 2025-04-29

**Authors:** Shunxin Xin, Zhongliang He, Weihua Xu, Xueming He, Guoxing Chen, Zhongrui Ye, Xia Hong

**Affiliations:** ^1^ Department of Thoracic Surgery Tongde Hospital of Zhejiang Province Hangzhou China; ^2^ Department of Respiratory and Critical Care Medicine Tongde Hospital of Zhejiang Province Hangzhou China; ^3^ Department of Cardiothoracic Surgical Nursing Tongde Hospital of Zhejiang Province Hangzhou China

**Keywords:** bronchopleural fistula, critically severe empyema, ECMO

## Abstract

Empyema is one of the most dangerous complications in thoracic surgery. It can easily progress to severe empyema with life‐threatening conditions that are extremely challenging to treat. We successfully treated a patient with critically severe empyema via extracorporeal membrane oxygenation (ECMO), and the patient eventually recovered and was discharged. A 64‐year‐old male developed empyema 5 years ago. The thoracic infection led to a breakdown of the left chest wall. During hospitalisation, the patient's condition gradually deteriorated. We promptly initiated ECMO support and performed multiple debridement surgeries, fistula closure, targeted anti‐infection treatment, and nutritional support. Ultimately, the patient's condition improved, as he eventually recovered and was discharged.

## Graphical abstract

Here, we report our treatment experience of a patient with critically severe empyema on extracorporeal membrane oxygenation (ECMO) and subsequent complex treatments, ultimately resulting in his recovery.

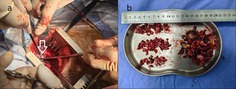

## 1 Introduction

Empyema is one of the most serious complications in thoracic surgery, presenting significant treatment challenges with a mortality rate of approximately 5.6%. It frequently coexists with bronchopleural fistulas, making treatment particularly challenging [1]. A subset of empyema patients progress to severe empyema due to inadequate pus drainage or delayed treatment, leading to critically ill conditions and even death. Therefore, the treatment of empyema presents significant challenges [2, 3, 4]. We treated a patient with critically severe empyema on ECMO and subsequent complex treatments, ultimately resulting in the recovery of the patient. We report our treatment experience as follows.

## 2 Case Report

A 64‐year‐old male underwent left thoracotomy and pneumonectomy for lung cancer 18 years ago, recovering well postoperatively. Five years ago, he developed a cough, haemoptysis, and bloody sputum without fever or chills, not seeking medical care. One year prior, he developed a left chest wall expanding lesion (1–6 cm) that ruptured, discharging rust‐coloured, foul‐smelling pus. This led to improvement in haemoptysis but occasional foul‐smelling sputum. Anti‐infection therapy at a local hospital showed no improvement. On admission in May 2023, the physical exam revealed left lung absence, a chest wall sinus tract, and pus discharge. He was diagnosed with left empyema with fistula and chest wall fistula.

On admission, the patient had a temperature of 38.2°C, WBC of 29.4 × 10^9^/L, neutrophil count of 24.7 × 10^9^/L, and CRP of 78.4 mg/L. Empirical moxifloxacin 0.4 qd IV and nutritional support were initiated. Chest tube drainage on Day 3 yielded 50 mL of pus daily, with no bacterial or fungal growth. Fever and blood counts persisted. Anaerobic infection was suspected, but the patient refused antibiotic changes. Fever and empyema progressed, leading to respiratory failure. Endotracheal intubation (Day 5) and double‐lumen intubation (Day 6) were performed, but significant air leakage from the bronchial stump compromised oxygenation.

VV‐ECMO support was initiated from Day 7 to Day 26. In VV‐ECMO, blood was drawn from the inferior vena cava through a catheter inserted into the femoral vein, and then perfused into the right atrium via a catheter placed in the internal jugular vein. The rotational speed of VV ECMO was set at 3000 rpm, and the blood flow rate was set at 3 L/min. The temperature of the water tank was maintained at 37.0°C (+0.5°C). Low‐molecular‐weight heparin was used for anticoagulation, and the APTT was maintained between 55 and 65 s.

The ventilator was set as follows: it operated in the pressure control mode, with a plateau pressure of 20 cm H_2_O, a fraction of inspired oxygen (FiO_2_) of 0.3, a positive end‐expiratory pressure (PEEP) of 10 cm H_2_O, a respiratory rate of 10 breaths per minute, and an inspiratory‐to‐expiratory (I:E) ratio of 1:1. As a result, oxygenation was stabilised. A Y‐shaped silicone stent (left side with a blind end) was placed on Day 8, significantly reducing air leakage. On Day 30, the medical team conducted a tracheotomy on the patient, aiming to streamline the process of respiratory tract care and enhance the overall efficacy of treatment.

The nutritional support plan was dynamically adjusted according to the patient's condition to correct the patient's hyperproteinaemia and anaemia. The plan was also adjusted dynamically based on the patient's blood albumin level and fever status.

Intravenous injections of Compound Amino Acid (18AA‐V‐SF) were given from Day 1 to Day 5, Day 58 to Day 67 and Day 88 to Day 96, and Structured Fat Emulsion (C6‐24) was administered from Day 58 to Day 67. Meanwhile, enteral nutritional preparations were orally taken to increase energy support and reduce the burden of infusion. Specifically, Enteral Nutritional Suspension (TPF) was given from Day 42 to Day 59, and Enteral Nutritional Emulsion (TP‐HE) was given from Day 69 to Day 96.

In addition, Human Albumin (20%) was injected from Day 60 to Day 67 to correct hyperproteinaemia, and Polysaccharide Iron Complex was orally administered to correct anaemia. Moreover, *Bacillus licheniformis* Capsules were given from Day 42 to Day 67 to promote nutrient absorption.

**FIGURE 1:**
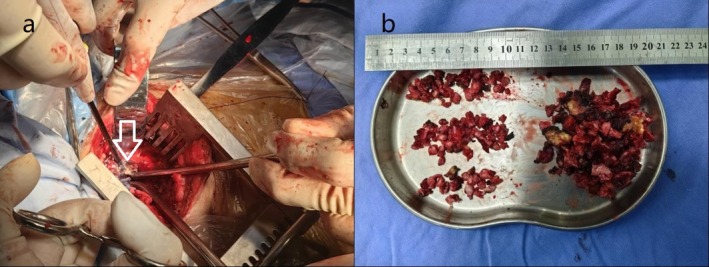
During surgery, thick, caseous purulent fluid was observed in the pleural cavity, which could not drain through the chest tube (a); the purulent debris and necrotic tissue were removed (b).

Systemic and empyema infections worsened, with CRP rising to 91.9 mg/L. mNGS of pus identified multiple bacterial species, including multidrug‐resistant *Acinetobacter baumannii* , *Klebsiella pneumoniae* , *Serratia marcescens* , *Pseudomonas fluorescens* and *Pseudomonas aeruginosa* , with resistance genes detected. After MDT discussion, the patient received omadacycline (Days 6–42), meropenem (Days 8–23) and ceftazidime‐avibactam (Days 28–42) for anti‐infection therapy, leading to improvement in blood counts and infection status. The patient was successfully weaned off the ventilator on Day 35. However, a CT scan on Day 43 showed a significant increase in left empyema fluid, indicating poor drainage. Surgery was indicated, and the patient underwent left empyema debridement and chest wall window surgery on Day 43, where a large amount of caseous pus was observed intraoperatively (Figure 1).

Postoperatively, persistent oozing and pus formation indicated poor local infection control. A second debridement surgery was performed on Day 52, with pus cultures negative for bacteria and fungi. Due to multidrug‐resistant *Pseudomonas aeruginosa* and *Klebsiella pneumoniae* in sputum cultures, meropenem and linezolid (Days 43–46) were administered initially, then de‐escalated to cefoperazone‐sulbactam (Days 46–63). Rehabilitation intervention improved swallowing function and skeletal muscle strength, leading to gradual recovery.

On Day 62, the patient developed a low‐grade fever, cough, and increased sputum production, with wet rales in the right lower lung. CT confirmed a right lung infection. The infectious disease department adjusted antibiotics to polymyxin B (Days 63–67), amikacin (Days 63–75) and meropenem (Days 68–85). Pulmonary infection and empyema improved, with reduced cough, sputum and improved nutritional status. Antibiotics were stopped. The patient's anaemia and hyperproteinaemia were well‐controlled, and swallowing function improved. The tracheostomy tube was removed on Day 151, and the patient transitioned to nasal breathing. Follow‐up at 270 days showed good recovery, controlled chest infection, 5 kg weight gain and a healed tracheostomy site.

**FIGURE 2:**
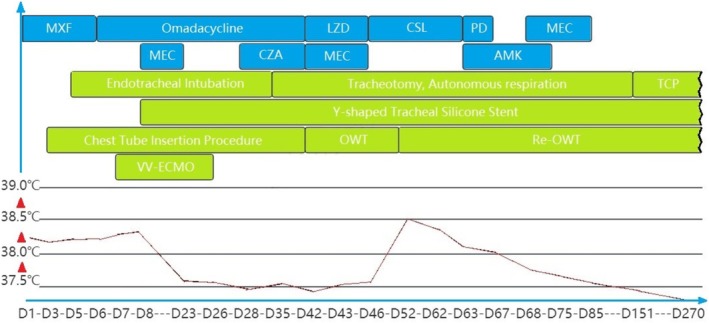
Schematic of the treatment process. AMK, amikacin; CSL, cefoperazone‐sulbactam; CZA, ceftazidime‐avibactam; LZD, linezolid; MEC, meropenem; MXF, moxifloxacin; OWT, open window thoracoscopic; PB, polymyxin B; Re‐OWT, reopen window thoracostomy; TCP, tracheostomy closure procedure; VV‐ECMO, venovenous extracorporeal membrane oxygenation.

The overall treatment process is detailed in Figure 2.

## 3 Discussion

Diagnosis and treatment of severe empyema, especially post‐ECMO, are challenging due to complex issues like severe infection, bronchopleural fistula and inadequate drainage [1, 2, 3, 4]. Key challenges include targeted anti‐infection therapy, cavity drainage maintenance and fistula sealing [5, 6, 7, 8]. Traditional antibiotic selection relies on pus culture and susceptibility testing, but low culture positivity rates can complicate treatment [9]. Metagenomic next‐generation sequencing (mNGS) rapidly detects all pathogens and resistance genes in clinical samples, guiding targeted antibiotic therapy [9]. In this case, the first culture was negative, but mNGS identified 13 bacteria, including multidrug‐resistant *Acinetobacter baumannii* , *Klebsiella pneumoniae* , *Serratia marcescens* , *Pseudomonas fluorescens* and *Pseudomonas aeruginosa* , improving infection control after drug adjustments.

Targeted anti‐infection treatment and timely adjustment of antibiotics are of great significance [6]. Initial empirical anti‐infection with moxifloxacin was given on Day 1. The mNGS of pus found multiple drug‐resistant bacteria. After MDT, omadacycline, meropenem and ceftazidime‐avibactam were used (Days 6–42, etc.), improving the condition. Due to resistant bacteria in sputum, treatment was adjusted. On Day 62, a new infection occurred. Antibiotics were readjusted. Finally, the infection improved and antibiotics were stopped.

Adequate empyema cavity drainage is critical, requiring techniques like closed drainage, debridement and window surgery [6]. In this case, ineffective chest tube drainage led to multiple debridements and window surgeries, improving infection control. Large bronchopleural fistulas (> 10 mm) complicate treatment, causing air leakage and hypoxia [7, 8]. The use of a Y‐shaped silicone stent to seal the fistula in this case significantly improved air leakage and oxygenation, aiding recovery.

ECMO's role in severe empyema with ARDS/cardiopulmonary failure is notable, providing a rescue window [10]. However, ECMO increases the risk of bleeding, infections and malnutrition [11]. Anticoagulation is necessary, but it can lead to haemoptysis or bleeding [11]. In this case, pleural cavity bleeding during anticoagulation was managed via debridement and infection control, successfully halting bleeding and improving infection status.

The nutritional support plan should be dynamically adjusted according to the patient's condition, blood albumin level and fever status. A comprehensive nutritional support strategy integrating multiple modalities, including oral administration and intravenous injection, along with the infusion of blood products (human albumin or red blood cells) when necessary, may be a suitable option. Intravenous injections of Compound Amino Acid (18AA‐V‐SF) were given on several occasions, and Structured Fat Emulsion (C6‐24) was administered from Days 58 to 67. Enteral nutritional preparations were taken orally. Human Albumin was injected to correct hyperproteinaemia, and Polysaccharide Iron Complex for anaemia. *Bacillus licheniformis* Capsules were given to aid absorption.

Catheter‐related infections are significant complications in empyema patients undergoing ECMO [11]. Thorough catheter disinfection, regular monitoring of infection markers and pus culture with antibiotic sensitivity testing are essential. Adequate drainage, regular disinfection and timely dressing changes are necessary [12, 13].

In this case of critically ill empyema, ECMO played a crucial role in rescuing the patient, providing irreplaceable support for maintaining the patient's life and securing valuable time for subsequent treatments. Although using ECMO in patients with BPF presents a unique set of challenges, it is feasible if certain aspects of ECMO are appropriately managed.

## Author Contributions

Zhongliang He, Weihua Xu and Xueming He: data curation. Shunxin Xin and Xia Hong: investigation. Guoxing Chen and Zhongrui Ye: methodology. Shunxin Xin and Xia Hong: writing, review and editing.

## Ethics Statement

The authors declare that appropriate written informed consent was obtained for the publication of this manuscript and accompanying images. The study involving human participant was reviewed and approved by the Ethics Committee of the Tongde Hospital of Zhejiang Province.

## Conflicts of Interest

The authors declare no conflicts of interest.

## Data Availability

The datasets generated during and/or analysed during the current study are available from the corresponding author on reasonable request.
